# Tanshinone IIA inhibits cardiomyocyte pyroptosis through TLR4/NF-κB p65 pathway after acute myocardial infarction

**DOI:** 10.3389/fcell.2023.1252942

**Published:** 2023-09-12

**Authors:** Ruoning Chai, Zelin Ye, Wenjing Xue, Shuqing Shi, Yi Wei, Yuanhui Hu, Huaqin Wu

**Affiliations:** Department of Cardiology, Guang’anmen Hospital, China Academy of Chinese Medical Sciences, Beijing, China

**Keywords:** tanshinone IIA, heart failure, myocardial infarction, pyroptosis, inflamation

## Abstract

**Background:** Tanshinone IIA, derived from Radix Salviae Miltiorrhizae (Salvia miltiorrhiza Bunge), constitutes a significant component of this traditional Chinese medicine. Numerous studies have reported positive outcomes regarding its influence on cardiac function. However, a comprehensive comprehension of the intricate mechanisms responsible for its cardioprotective effects is still lacking.

**Methods:** A rat model of heart failure (HF) induced by acute myocardial infarction (AMI) was established via ligation of the left anterior descending coronary artery. Rats received oral administration of tanshinone IIA (1.5 mg/kg) and captopril (10 mg/kg) for 8 weeks. Cardiac function was assessed through various evaluations. Histological changes in myocardial tissue were observed using staining techniques, including Hematoxylin and Eosin (HE), Masson, and transmission electron microscopy. Tunel staining was used to detect cell apoptosis. Serum levels of NT-pro-BNP, IL-1β, and IL-18 were quantified using enzyme-linked immunosorbent assay (ELISA). Expression levels of TLR4, NF-κB p65, and pyroptosis-related proteins were determined via western blotting (WB). H9C2 cardiomyocytes underwent hypoxia-reoxygenation (H/R) to simulate ischemia-reperfusion (I/R) injury, and cell viability and apoptosis were assessed post treatment with different tanshinone IIA concentrations (0.05 μg/ml, 0.1 μg/ml). ELISA measured IL-1β, IL-18, and LDH expression in the cell supernatant, while WB analysis evaluated TLR4, NF-κB p65, and pyroptosis-related protein levels. NF-κB p65 protein nuclear translocation was observed using laser confocal microscopy.

**Results:** Tanshinone IIA treatment exhibited enhanced cardiac function, mitigated histological cardiac tissue damage, lowered serum levels of NT-pro-BNP, IL-1β, and IL-18, and suppressed myocardial cell apoptosis. Moreover, tanshinone IIA downregulated the expression of TLR4, NF-κB p65, IL-1β, pro-IL-1β, NLRP3, Caspase-1, and GSDMD-N pyroptosis-related proteins in myocardial tissue. Additionally, it bolstered H/R H9C2 cardiomyocyte viability, curbed cardiomyocyte apoptosis, and reduced the levels of TLR4, NF-κB p65, IL-1β, pro-IL-1β, NLRP3, Caspase-1, and GSDMD-N pyroptosis-related proteins in H/R H9C2 cells. Furthermore, it hindered NF-κB p65 protein nuclear translocation.

**Conclusion:** These findings indicate that tanshinone IIA enhances cardiac function and alleviates myocardial injury in HF rats following AMI. Moreover, tanshinone IIA demonstrates potential suppression of cardiomyocyte pyroptosis. These effects likely arise from the inhibition of the TLR4/NF-κB p65 signaling pathway, presenting a promising therapeutic target.

## 1 Introduction

Heart failure (HF) marks the advanced phase of diverse cardiovascular disorders and impacts approximately 64.3 million individuals globally (Lippi and Sanchis-Gomar, 2020). Once HF manifests, its reversal becomes formidable, leading to a notable deterioration in patients’ quality of life. Investigations have approximated that 1%–2% of adults in developed nations are afflicted with diagnosed HF(Sahle et al., 2016; Groenewegen et al., 2020). The survival rates for HF patients at 1, 2, 5, and 10 years stand at around 87%, 73%, 57%, and 35%, correspondingly, with HF incidents amplifying mortality risk fivefold (Jones et al., 2019). Despite considerable strides in HF treatment, clinical outcomes continue to be unsatisfactory, particularly in terms of enhancing patients’ quality of life (Freedland et al., 2021). Research has indicated that within a year of discharge post acute myocardial infarction (AMI), about 20%–30% of patients develop HF(Jenča et al., 2020). Hence, exploration into drugs that shield cardiac function following AMI holds substantial promise.

Cardiac remodeling emerges as a pivotal pathological mechanism in the initiation and advancement of HF, with myocardial cell demise constituting a central process in this remodeling (Mishra et al., 2019). Pyroptosis, a variant of programmed cell demise, maintains close ties with physiological and pathological heart processes (Gao et al., 2022). Detection of pyroptosis signals hinges on pattern recognition receptors, encompassing NOD-like receptors (NLR), Toll-like receptors (TLR), and C-type lectin receptors (CLR) (Yang et al., 2018; Zhou et al., 2020; Jin et al., 2022). Among these, TLR plays a pivotal role in instigating the signal cascade, culminating in cell activation and inflammatory cytokine generation (Swanson et al., 2020). Particularly, TLR4 becomes activated under myocardial ischemic hypoxia conditions (Zhao et al., 2009). Once activated, TLR4 incites NF-κB p65 activation, subsequently triggering NLRP3 inflammasome activation, leading to Caspase-1 activation (Liu et al., 2017; Yang et al., 2020). The activated Caspase-1 cleaves Gasdermin D (GSDMD), yielding a GSDMD nitrogen terminal activity domain (GSDMD-N) peptide fragment that triggers cell membrane perforation, rupture, and content release, instigating pyroptosis (Liu et al., 2016a; Tsuchiya et al., 2019). Additionally, Caspase-1 cleaves IL-1β and IL-18 precursors to form their active forms (Sansonetti et al., 2000; Xia et al., 2021), which are then extracellularly released, attracting inflammatory cells and exacerbating the inflammatory response. Under normal conditions, pyroptosis is maintained at a low baseline level, contributing to cellular equilibrium (Yu et al., 2021). However, during ischemia or hypoxia, excessive pyroptosis activation, coupled with sustained inflammatory reactions, accelerates adverse cardiac remodeling and deteriorates heart function (Toldo et al., 2018; Zeng et al., 2019). Thus, drug exploration that centers on the TLR4/NF-κB p65 pathway to hinder myocardial pyroptosis and enhance cardiac function holds considerable potential.

Radix Salviae Miltiorrhizae (Salvia miltiorrhiza Bunge) is a conventional Chinese medicinal herb renowned for its varied therapeutic effects, encompassing pain alleviation, activation of blood circulation, elimination of blood stasis, heart clearance, cooling of blood, and elimination of carbuncles, aligned with traditional Chinese medicine principles (Zhou et al., 2019; Fang et al., 2020). It has gained widespread use for its cardiovascular benefits, including antioxidant, anti-inflammatory, and antifibrotic attributes (Su et al., 2015; Fang et al., 2018a; Jung et al., 2020). A clinical trial has documented that a 3-month regimen of salvia miltiorrhiza compounds can diminish coronary heart disease risk by ameliorating blood lipid levels (Liu et al., 2016b). The primary active components in Radix Salviae Miltiorrhizae comprise phenolic acids and tanshinones. Among these, Tanshinone IIA has been extensively researched for its biological activities, exhibiting promising results in treating atherosclerosis, cardiac hypertrophy, enhancing heart function, alleviating cerebral ischemia, and presenting potential advantages for Alzheimer’s disease. These effects are closely tied to its anti-inflammatory and antioxidant properties, capacity to impede cell apoptosis, and role in safeguarding mitochondrial function (Gao et al., 2012; Guo et al., 2020; Ansari et al., 2021; Subedi and Gaire, 2021; Wang et al., 2022; Wang and Wu, 2022). Recent investigations have demonstrated that tanshinone IIA can amplify the mitigation of mesenchymal stem cell-derived exosome-induced myocardial ischemia-reperfusion (I/R) injury by elevating miR-223-5p (Li et al., 2023). It can also ameliorate cardiac hypertrophy through galectin-3, suppress cardiomyocyte apoptosis (Zhang et al., 2022), and preserve cardiac function during doxorubicin-triggered cardiac toxicity (Xu et al., 2022). However, present research has not yet explored whether tanshinone IIA is implicated in pyroptosis. In this study, we employed a left anterior descending coronary artery (LADCA) ligation-induced HF rat model and an *in vitro* hypoxia-reoxygenation (H/R)-stimulated H9C2 cardiomyocyte model to delve into the potential mechanism of tanshinone IIA in hindering myocardial pyroptosis and ameliorating HF and cardiac remodeling via the TLR4/NF-κB p65 pathway.

## 2 Materials and methods

### 2.1 Drugs and reagents

Tanshinone IIA (B20257, HPLC≥98%, China), captopril (H32023731, China), Dulbecco’s Modified Eagle Medium (D6570, Solarbio, China), streptomycin (T1320, Solarbio, China), penicillin (P1400, Solarbio, China), fetal bovine serum (10099141, Gibco, United States), anti-NF-κB p65 (ab76302, Abcam, United Kingdom), anti-NLRP3 (ab263899, Abcam, United Kingdom), anti-pro-Caspase1 (ab179515, Abcam, United Kingdom), anti-Caspase1 (ab138483, Abcam, United Kingdom), anti-GSDMD (ab209845, Abcam, United Kingdom), anti-TLR4 (19811-1-Ap, proteintech, China), anti-IL-1β (16806-1-AP, proteintech, China), anti-GAPDH (60004-1-Ig, proteintech, China), anti-β-actin (66009-1-Ig, proteintech, China), anti-pro-IL-1β (WL02257, Wanleibio, China), BNP (ab108815, Abcam, United Kingdom), IL-1β (ab255730, Abcam, United Kingdom), IL-18 (ab213909, Abcam, United Kingdom), TUNEL Apoptosis Assay Kit (KGA7071, China).

### 2.2 Animals and ethics statement

Male Sprague-Dawley (SD) rats, aged 6 weeks and weighing 180–200 g, were obtained from Beijing Hua Fu Kang Bioscience Co., LTD. The rats were housed under standardized conditions, including a 12-h light/dark cycle and a temperature of 22°C ± 2°C. They were provided *ad libitum* access to food and water throughout the experiment. All experimental procedures and animal welfare practices strictly adhered to the Ethical Regulations on the Care and Use of Laboratory Animals of Guang’anmen Hospital to ensure ethical treatment of the animals (IACUC-GAMH-2021-020).

### 2.3 Experimental protocol

This study encompassed a total of 100 male Sprague-Dawley (SD) rats. The LADCA ligation technique was employed to establish the AMI model. The rats were anesthetized with intraperitoneal sodium pentobarbital (50 mg/kg) and subjected to chest opening for heart exposure. The LADCA was ligated using a 5-0 surgical suture. Post-operation, the rats were placed on an electric blanket for temperature maintenance until regaining consciousness.

For the sham group, the LADCA was threaded but not ligated, while all other surgical steps remained consistent. At 24 h after the operation, rats in the LADCA ligation group were randomly allocated to three subgroups: the model group, tanshinone IIA group, and captopril group. Guided by prior research, the tanshinone IIA group received an optimal dosage of 1.5 mg/kg of tanshinone IIA (Zhang et al., 2019). The captopril group was administered captopril at 10 mg/kg dosage, based on body surface area equivalence between animals and humans, as per recommended daily human dosage. The sham and model groups received purified water. Drugs were orally administered once daily for an 8-week duration.

### 2.4 Echocardiography

At the 8-week mark post ligation, echocardiography was conducted under anesthesia. Rats were appropriately positioned after shaving their thoracic walls. Two-dimensional and M-mode echocardiography assessed cardiac morphology and function. An 8-MHz transducer connected to an HP5500 color Doppler ultrasound imaging instrument (Agilent, California, US) facilitated the procedure.

Echocardiography captured several parameters, including left ventricular end-diastolic diameter (LVIDd), left ventricular end-systolic diameter (LVIDs), fractional shortening (FS), and ejection fraction (EF), with the values averaged from three cardiac cycles.

### 2.5 Histological examination

Harvested myocardial tissues underwent 4% paraformaldehyde fixation for 30 h and storage at 4°C. Fixed tissues were paraffin-embedded and sliced into 6 μm-thick sections. Sections underwent deparaffinization, dehydration via xylene and ethanol gradient immersion, followed by staining procedures involving Hematoxylin and Eosin (HE), Masson’s trichrome staining, and Tunel staining (Sheng et al., 2019).

To prepare samples for transmission electron microscopy, left ventricular myocardium was diced into 1 mm^3 cubes and treated with 2.5% glutaraldehyde for 24 h at 4°C. Subsequently, samples were immersed in 1% osmium tetroxide, dehydrated with graded ethanol solutions, and embedded. 60 nm ultrathin slices of embedded specimens were double-stained with uranyl acetate and lead citrate before observation using a transmission electron microscope (H-7650, Hitachi Limited, Japan).

### 2.6 Measurement of inflammatory cytokines and HF biomarker

After drawing blood samples from the rat abdominal aorta, the samples were subjected to centrifugation at 3,000 rpm and 4°C for 10 min. This centrifugation process separated the blood into two components: plasma and cell supernatant. The plasma and cell supernatant were collected and stored at −80°C for further analysis.

To measure the levels of N-terminal pro-B-type natriuretic peptide (NT-pro-BNP), interleukin-1β (IL-1β), and interleukin-18 (IL-18), an enzyme-linked immunosorbent assay (ELISA) was employed. ELISA is a commonly used laboratory technique that allows for the quantification of specific proteins or molecules in a sample. In this case, the plasma samples and cell supernatant were analyzed using ELISA kits designed to detect and measure the concentrations of NT-pro-BNP, IL-1β, and IL-18. The ELISA procedure involves the use of specific antibodies that bind to the target molecules, allowing for their detection and quantification based on colorimetric or fluorescent signals.

### 2.7 Cell culture

H9C2 cells, obtained from the Chinese Academy of Medical Sciences (Institute of Basic Medicine, Beijing, China), were cultured in Dulbecco’s Modified Eagle’s Medium (DMEM) supplemented with 10% fetal bovine serum (FBS) and 1% penicillin-streptomycin (Gibco) at 37°C under 95% humidity and 5% CO2. The culture medium was refreshed every 2 days to support optimal cell growth and viability.

Prior to experiments, H9C2 cells were divided into four groups:

Control group: Cells cultured under standard conditions at 37°C, 95% humidity, and 5% CO2, serving as the baseline control.

Hypoxia/Reoxygenation (H/R) group: Cells subjected to 6 h of hypoxia followed by 24 h of reoxygenation to mimic ischemia-reperfusion injury.

Tanshinone IIA group: Cells treated with tanshinone IIA at concentrations of 0.05 μg/ml and 1 μg/ml for 24 h before reoxygenation.

This categorization enabled the assessment of tanshinone IIA’s effects on cells under normal conditions and during hypoxia/reoxygenation, relevant to cardiac ischemia-reperfusion injury.

### 2.8 Measurement of cardiomyocyte viability

After reaching over 90% confluence, H9C2 cells were detached from culture dishes using 0.25% trypsin (Sigma, St. Louis, MO, United States) for 3 min. The trypsin reaction was stopped with DMEM. Cell counting determined viable cell numbers.

Subsequently, H9C2 cells were seeded into 12-well plates at 50,000 cells per well, allowing adherence for 24 h in DMEM supplemented with 10% FBS and 1% penicillin-streptomycin at 37°C under 95% humidity and 5% CO2.

Cell viability was assessed using the Cell Counting Kit-8 (CCK-8) method, involving CCK-8 reagent addition to produce a colorimetric reaction in viable cells, generating a measurable signal reflecting cell viability. Absorbance or optical density was measured using a microplate reader for quantitative assessment.

### 2.9 Apoptosis detection

After 24-h treatment, adherent and detached H9C2 cells were collected. Apoptosis assessment involved staining collected cells with Annexin V-FITC and propidium iodide (PI) using a commercially available apoptosis detection kit. Annexin V binds to exposed phosphatidylserine on apoptotic cell membranes, while PI stains DNA in cells with compromised membranes.

Stained cells were analyzed using flow cytometry (BD C6 instrument) for apoptosis evaluation.

### 2.10 Western blotting

Proteins were extracted from both heart tissues and cells of each experimental group and subjected to SDS-PAGE for separation. The isolated proteins were then transferred onto polyvinylidene fluoride (PVDF) membranes under controlled conditions (250mA, 4°C, 90 min). Subsequently, these membranes were immersed in a TBST solution containing 5% non-fat milk (used as a blocking agent) and left to incubate for 2 h at room temperature. Primary antibodies specific to TLR4, NF-κB p65, IL-1β, pro-IL-1β, NLRP3, Caspase-1, pro-Caspase-1, and GSDMD were prepared in diluted form and applied to the PVDF membranes, which were then incubated at 4°C overnight. Following this, the membranes were thoroughly washed to remove unbound antibodies. The next step involved applying HRP-conjugated secondary antibodies, also diluted in the blocking solution. The membranes were incubated with these secondary antibodies at 37°C for 2 h. A second thorough washing of the membranes was performed to remove any unbound secondary antibodies. The membranes were then treated with a freshly prepared ECL reagent, a 1:1 mixture of enhanced solution and stable peroxide solution. For detecting protein bands, the membranes were exposed to film for 60 s using an eBlot exposure instrument. The optimal exposure time was determined, and images were captured and subsequently analyzed using ImageJ software.

### 2.11 The nuclear translocation of p65 was observed via confocal microscopy

Following treatment, the H9C2 cardiomyocytes were fixed, permeabilized, and blocked. Subsequently, they underwent overnight incubation at 4°C with primary antibodies targeting NF-κB p65, diluted at 1:500. After thorough triple washing with PBST, the cells were exposed to secondary antibodies labeled with FITC, diluted at 1:200. Nuclear counterstaining was then performed in a dark environment. Finally, the cells were examined using a laser confocal microscope.

### 2.12 Statistical analysis

All experimental data are presented as mean ± standard error of the mean. Comprehensive evaluations were conducted using analysis of variance (ANOVA). For comparisons between two independent groups, a t-test was employed. In the case of multiple groups, either a one-way or two-way ANOVA, followed by a Tukey post-hoc test, was utilized. Statistical significance was determined with a *p*-value less than 0.05.

## 3 Results

### 3.1 Effect of tanshinone IIA on cardiac function

**FIGURE 1:**
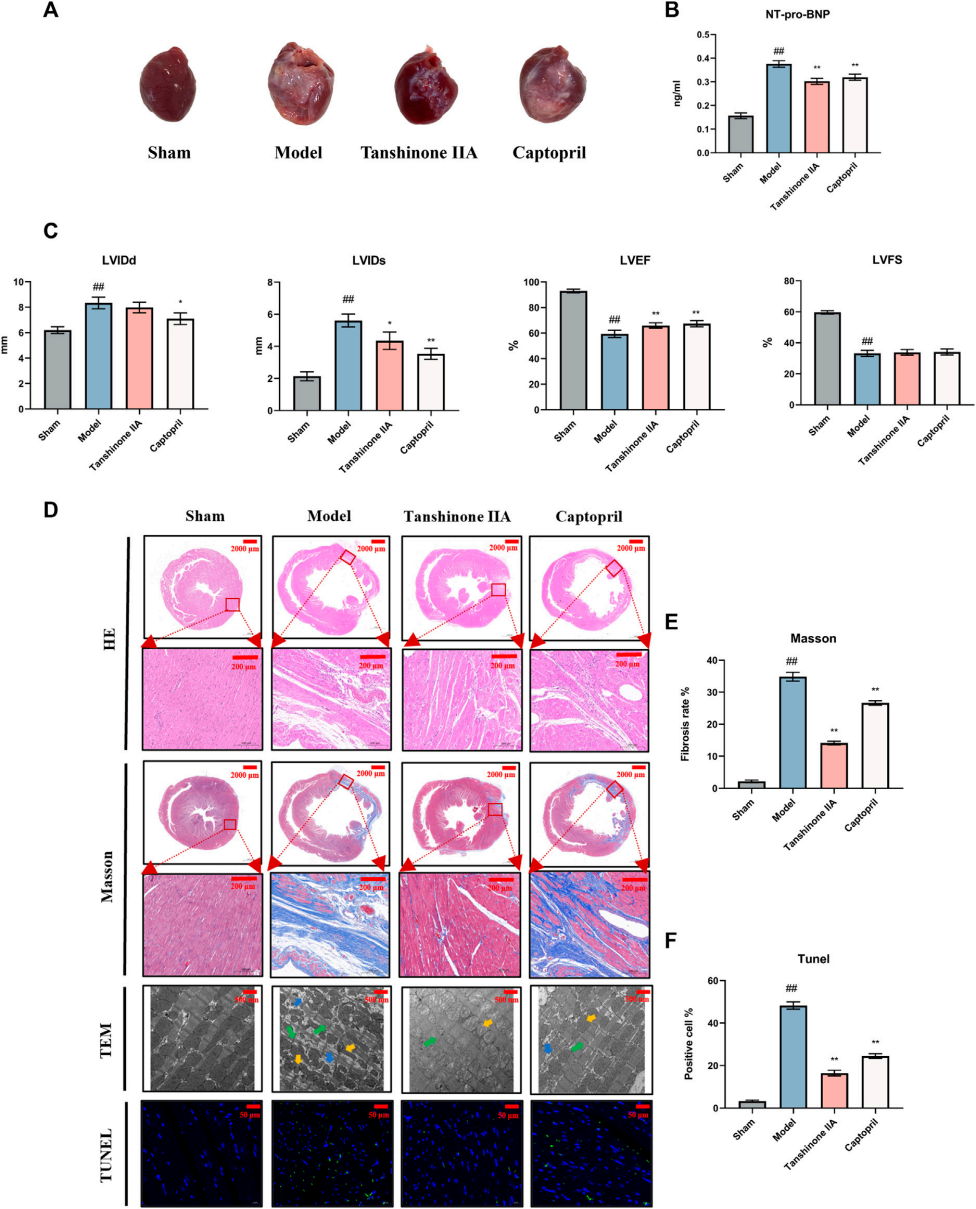
Effect of Tanshinone IIA on cardiac function in rats with heart failure after acute myocardial infarction. **(A)** Appearance of the hearts of each group of rats. **(B)** The expression of NT-pro-BNP in serum of rats in each group was detected. **(C)** Quantification of essential cardiac function parameters including left ventricular end-diastolic diameter (LVIDd), left ventricular end-systolic diameter (LVIDs), left ventricular ejection fraction (LVEF), and left ventricular fractional shortening (LVFS). These indicators serve as valuable tools to ascertain the efficacy of Tanshinone IIA in counteracting the HF on cardiac function. **(D)** The figure presents representative images from H&E staining (Scale bars = 2000–200 μm) provides an overall view of cellular integrity and layout, Masson’s Trichrome staining (Scale bars = 2000–200 μm) highlights the collagen deposits characteristic of fibrosis, TUNEL staining (Scale bars = 50 μm) allows the identification of apoptosis, and electron microscopy (Scale bars = 500 μm) offers an in-depth view of the ultrastructural changes in cardiac tissues. Together, these analyses reveal the promising potential of Tanshinone IIA in mitigating fibrosis and cellular damage after AMI. Electron microscopy arrow definition: The green arrows indicate the dark zone is characterized by areduction in the number and diameterof the cardiomyocytes. The yellow arrows indicate mitochondrial morphological atrophy, distortion, and deformation. The blue arrows mark the increase ofintracellular glycogen precipitation. **(E, F)** The semiquantitative analysis results of Masson’s trichrome staining and TUNEL staining. #*p* < 0.05 and ##*p* < 0.01 VS. the control group; *p < 0.05 and **p < 0.01 VS. the model group.

At the conclusion of the 8-week post-surgery period, during which heart samples were collected, external examination of the hearts revealed distinct differences. The model group exhibited a pale left ventricular anterior wall and an enlarged heart cavity compared to the sham surgery group. However, treatment with tanshinone IIA and captopril led to improvements in these visual characteristics (Figure 1A).

Echocardiography findings highlighted significant distinctions between the groups. The model group displayed increased LVIDd and LVIDs, alongside decreased LVEF and LVFS compared to the sham surgery group. Notably, intervention with tanshinone IIA and captopril resulted in reductions in LVIDd and LVIDs (*p* < 0.05), while leading to improved LVEF (*p* < 0.01) and LVFS. These observations underscore the presence of left ventricular systolic and diastolic dysfunction, as well as enlargement of the left ventricular cavity post-AMI. Both tanshinone IIA and captopril exhibited protective effects on cardiac function post-AMI (Figure 1C).

Analysis of the HF biomarker, plasma NT-pro-BNP, demonstrated significantly elevated levels in the model group in comparison to the sham surgery group. However, following tanshinone IIA intervention, NT-pro-BNP levels notably decreased, further affirming the protective role of tanshinone IIA in HF (Figure 1B).

Histological examination using HE staining revealed that cardiomyocytes in the sham group displayed organized arrangement, uniform shape, and consistent size. In contrast, the model group exhibited a substantial reduction in cardiomyocytes, disarray in muscle fiber alignment, and extensive infiltration of inflammatory cells. Treatment with tanshinone IIA and captopril led to reduced infarct size and alleviated myocardial tissue lesions compared to the model group (Figure 1D).

Analysis via Masson’s staining unveiled that the myocardial tissue in the infarct area of the model group had been replaced by collagen fibers. The marginal area of the infarct showed a loose cellular arrangement with abundant collagen fiber deposition in the intercellular space. Nevertheless, following treatment with tanshinone IIA and captopril, the severity of myocardial tissue lesions exhibited noticeable reduction in comparison to the model group (Figures 1D, E).

TUNEL staining outcomes indicated a substantial decrease in viable cell count within the model group as opposed to the sham group. However, administration of tanshinone IIA and captopril led to an increase in viable cell count (Figures 1D, F).

Examination under transmission electron microscopy unveiled severe myocardial structural damage within the model group. This damage was characterized by loose and disordered alignment of myocardial fibers, irregular morphology, and instances of fracture. Additionally, mitochondrial contraction, distortion, deformation, and glycogen accumulation were observed within cardiomyocytes. Nevertheless, after tanshinone IIA and captopril treatment, notable improvement in the alignment of myocardial fibers was observed in comparison to the model group (Figure 1D).

### 3.2 Tanshinone IIA enhances viability and reduces apoptosis in H/R H9C2 cardiomyocytes

**FIGURE 2:**
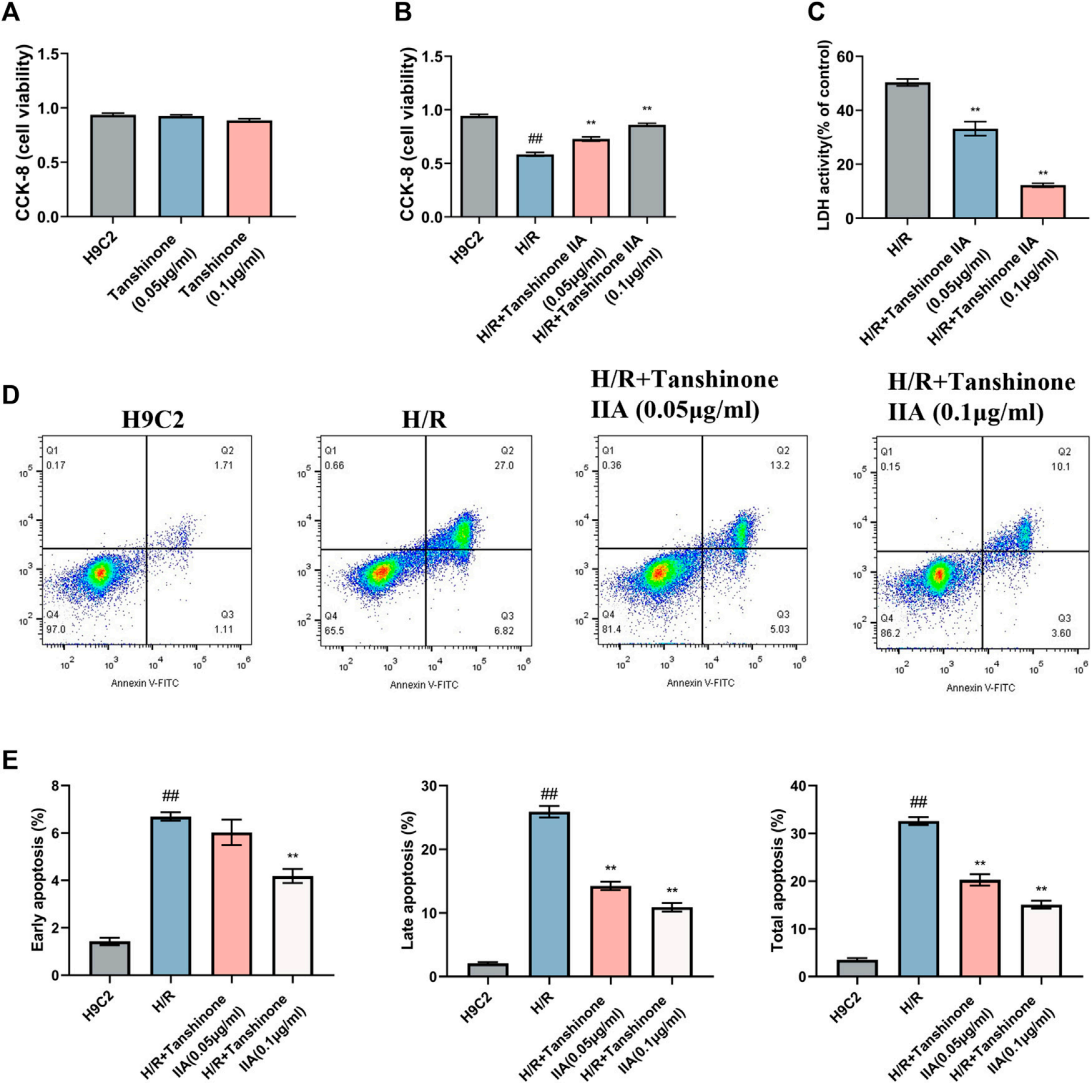
Impact of Tanshinone IIA on H/R H9C2 Cardiomyocytes. **(A)** Safety evaluation of Tanshinone IIA was conducted using CCK-8 assays to assess the effect on cell viability under normal conditions after 24 h of treatment. **(B)** Efficacy evaluation of Tanshinone IIA was conducted using CCK-8 assays to examine changes in cell viability in model conditions and post-intervention with Tanshinone IIA (0.05 μg/ml, 0.1 μg/ml). **(C)** LDH release assay to assess cellular injury following H/R insult. **(D)** Flow cytometry analysis, with four quadrants denoting live cells (Q4), early apoptotic cells, late apoptotic cells, and necrotic cells, offering a comprehensive view of the cellular state. **(E)** Bar chart displaying the percentages of early, late, and total apoptotic cells, highlighting the mitigatory effect of Tanshinone IIA on H/R-induced cellular apoptosis. #*p* < 0.05 and ##*p* < 0.01 VS. the control group; *p < 0.05 and **p < 0.01 VS. the model group.

The present study investigated the effects of tanshinone IIA on cell viability, apoptosis, and pyroptosis in H9C2 cardiomyocytes exposed to H/R. Intervention with tanshinone IIA demonstrated a significant improvement in the proliferative capacity of H9C2 cells, as indicated by CCK-8 assays after a 24-h incubation period. These results highlight the favorable proliferative effect and safety profile of tanshinone IIA under normal conditions (Figure 2A). Moreover, tanshinone IIA intervention led to a notable increase in cell viability compared to the model group following H/R (Figure 2B). This intervention resulted in enhanced cell survival rates and reduced apoptosis indices. Additionally, lactate dehydrogenase (LDH) measurements, a marker for cellular injury, indicated that tanshinone IIA effectively mitigated H/R-induced cell damage (Figure 2C). Flow cytometry analysis provided further evidence of reduced apoptosis, confirming the potential cardioprotective effects of tanshinone IIA (Figures 2D, E).

### 3.3 Tanshinone inhibits TLR4/NF-κB p65 signaling pathway and pyroptosis in rats with HF after AMI

**FIGURE 3:**
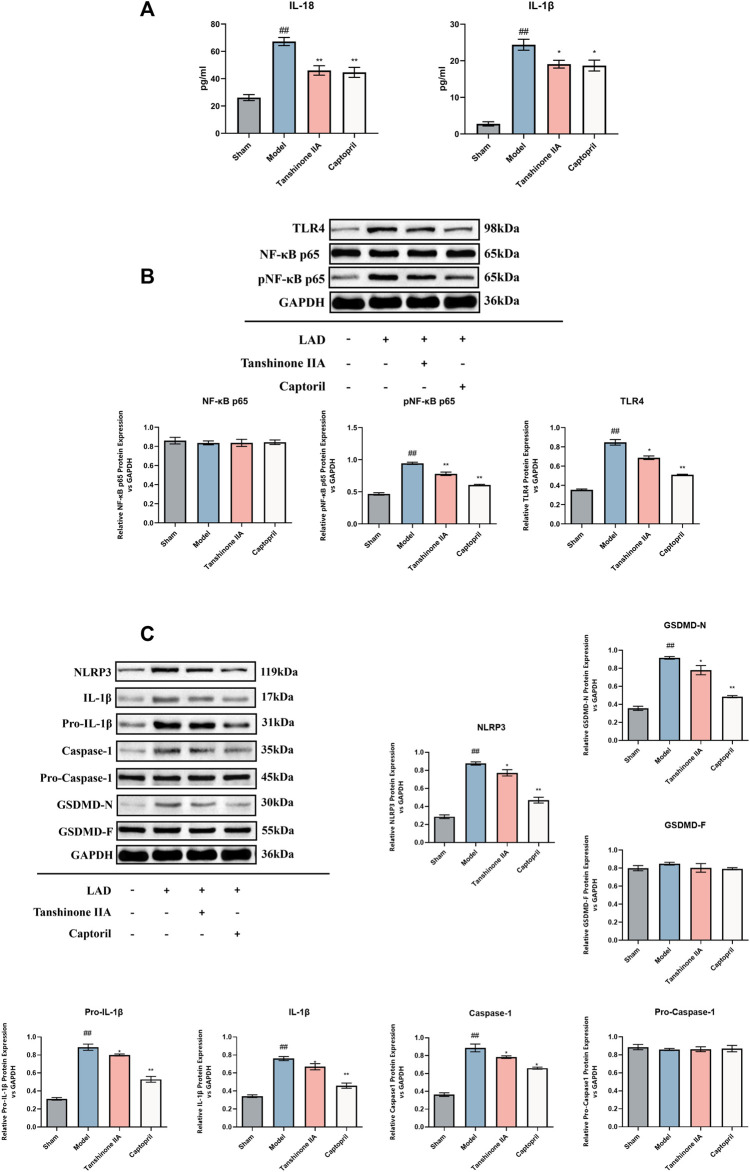
The Effect of Tanshinone IIA on the TLR4/NF-κB p65 Signaling Pathway and Pyroptosis in HF Rats. **(A)**: Detection of the inflammatory cytokines IL-18 and IL-1β was performed using ELISA. **(B)**: The levels of TLR4, NF-κBp65, and p-NF-κBp65 were determined via Western blot analysis. **(C)**: Western blot was also used to examine the levels of NLRP3, IL-1β, pro-IL-1β, Caspase1, pro-Caspase1, and GSDMD-N/F. #*p* < 0.05 and ##*p* < 0.01 VS. control group; *p < 0.05 and **p < 0.01 VS. model group. The figure demonstrates the considerable inhibitory effect of the Tanshinone IIA on these signaling pathways and proteins related to pyroptosis.

The current study revealed that tanshinone IIA demonstrated inhibitory effects on inflammatory cytokines and the TLR4/NF-κB p65 signaling pathway in cardiomyocytes, thereby suppressing cardiomyocyte pyroptosis in rats with HF following AMI. In comparison to the sham group, the model group exhibited a notable increase in IL-1β and IL-18 levels (*p* < 0.05, Figure 3A). Furthermore, there was an elevated expression of TLR4 and phosphorylated NF-κB p65 (pNF-κB p65) in cardiomyocytes, showing statistical significance (*p* < 0.05). However, intervention with captopril and tanshinone IIA resulted in a significant reduction of IL-1β, IL-18, TLR4, and phosphorylated NF-κB p65 (*p* < 0.05, Figure 3B). Additionally, the levels of NLRP3, Caspase-1, IL-1β, pro-IL-1β, and GSDMD-N were notably elevated in the model group (*p* < 0.05). In contrast, treatment with captopril and tanshinone IIA significantly downregulated these markers (*p* < 0.05, Figure 3C).

### 3.4 Tanshinone IIA inhibits pyroptosis in H/R H9C2 cardiomyocytes

**FIGURE 4:**
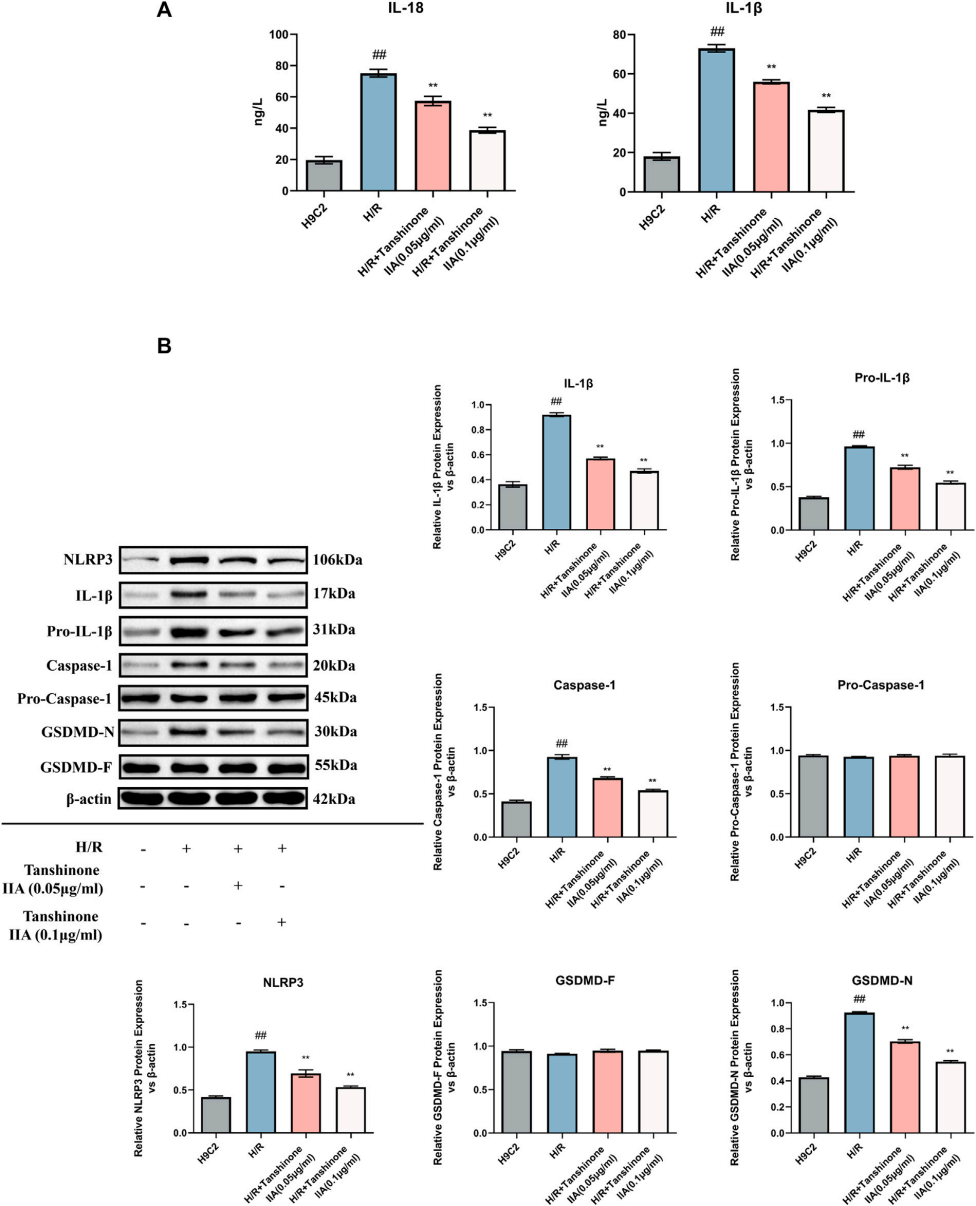
Impact of Tanshinone IIA on Pyroptosis. **(A)** Expression levels of the cytokines IL-18 and IL-1β were examined using ELISA. **(B)** Western blot was used to assess the expression levels of key pyroptosis markers: NLRP3, IL-1β, pro-IL-1β, Caspase1, pro-Caspase1, and GSDMD-N/F. #*p* < 0.05 and ##*p* < 0.01 VS. control group; *p < 0.05 and **p < 0.01 VS. model group. This indicates the significant downregulation of these markers in response to Tanshinone IIA intervention.

In addition to its positive impact on cell viability and apoptosis, tanshinone IIA also exhibited promising effects in mitigating pyroptosis in H/R-exposed H9C2 cardiomyocytes. ELISA results indicated a significant reduction in IL-18 and IL-1β, two critical cytokines released during pyroptosis, upon treatment with tanshinone IIA (*p* < 0.01, Figure 4A). Moreover, western blot analysis revealed a noticeable decrease in the expression levels of NLRP3, Caspase-1, IL-1β, pro-IL-1β, and GSDMD-N, pivotal proteins in the pyroptosis pathway and essential markers (*p* < 0.01, Figure 4B). These findings affirm tanshinone IIA’s inhibitory effect on pyroptosis, underscoring its potential as a therapeutic agent for H/R-exposed H9C2 cardiomyocytes.

### 3.5 Tanshinone IIA inhibits the TLR4/NF-κB p65 signaling pathway

**FIGURE 5:**
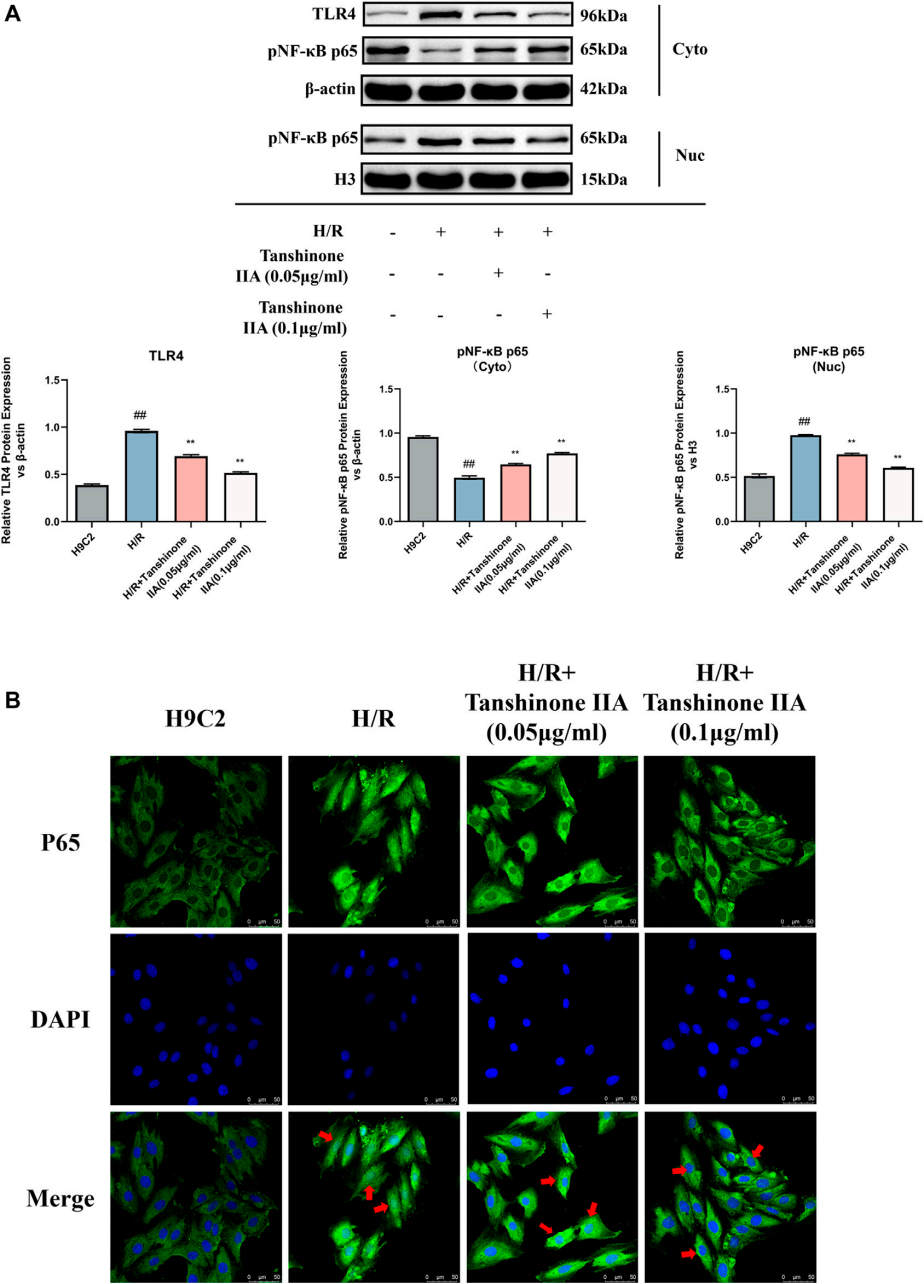
The impact of Tanshinone IIA on the TLR4/NF-κBp65 pathway. **(A)** Western blot was used to assess the expression of TLR4 in the cytosol, and the expression of NF-κBp65 in both the cytosol and the nucleus. **(B)** The nuclear translocation of p65 was observed via confocal microscopy. #*p* < 0.05 and ##*p* < 0.01 VS. control group; *p < 0.05 and **p < 0.01 VS. model group. These significant variations denote the modulatory effects of Tanshinone IIA on the TLR4/NF-κBp65 pathway.

The Western blot analysis results showed a notable reduction in the expression levels of TLR4 and pNF-κB p65 in the cytoplasm following tanshinone IIA intervention compared to the model group (*p* < 0.05, Figure 5A). Conversely, there was a significant increase in the expression of pNF-κB p65 in the nucleus (*p* < 0.05, Figure 5A). Immunofluorescence analysis further substantiated the effective inhibition of NF-κB p65 activity and nuclear translocation by tanshinone IIA intervention (Figure 5B).

## 4 Discussion

This study delved into the protective role of tanshinone IIA in cardiac health and its potential to curtail cardiomyocyte pyroptosis through a combination of *in vivo* and *in vitro* experiments. Our key findings are as follows: ① Tanshinone IIA improves cardiac function and myocardial structure in rats following AMI leading to HF. This enhancement is evident in improved cardiac function metrics and reduced myocardial tissue damage. ② Tanshinone IIA effectively suppresses cardiomyocyte pyroptosis. This is manifest in diminished pyroptotic markers and decreased levels of inflammatory cytokines linked to pyroptosis. ③ The inhibition of cardiomyocyte pyroptosis by tanshinone IIA is possibly mediated via the TLR4/NF-κB p65 signaling pathway. This treatment downregulates TLR4 and pNF-κB p65 expression and hampers NF-κB p65 nuclear translocation.

Tanshinone IIA, utilized in this study, has demonstrated its therapeutic potential in bolstering cardiac function. It has emerged as a prominent therapeutic agent in the management of cardiovascular diseases, with well-established efficacy. The favorable effects of tanshinone IIA manifest through diverse physiological pathways. It functions as a regulator of platelet aggregation (Wang et al., 2020a), thereby diminishing the risk of clot formation. Furthermore, its anti-inflammatory properties mitigate the inflammatory responses commonly linked to heart conditions (Lu et al., 2022). Tanshinone IIA also plays a significant role in energy metabolism (Li et al., 2020; Liu et al., 2021; Qi et al., 2022), which is crucial for maintaining the high-energy demand of the heart. Its additional cardiovascular effects include antioxidant activity (Yan et al., 2021), anti-atherosclerotic effects (J. Wang et al., 2020a; Wang et al., 2020b), protection against myocardial I/R injury (Li et al., 2023; Zhu et al., 2023), regulation of lipid metabolism (Gao et al., 2021), anti-fibrotic effects (Yan et al., 2022), vasodilation (Fan et al., 2011), and potential anti-arrhythmic effects (Shan et al., 2009), further demonstrating its multi-faceted role in promoting cardiovascular health.

Our findings, derived from cardiac ultrasound and BNP measurements, indicate that tanshinone IIA possesses the capacity to enhance cardiac function in rats with HF induced by AMI. Moreover, histological assessments encompassing HE staining, Masson’s staining, Tunel staining, and transmission electron microscopy unveil tanshinone IIA’s potential to ameliorate myocardial fibrosis and mitigate cardiomyocyte apoptosis. Furthermore, we evaluated the expression levels of pivotal pyroptosis-related proteins, including NLRP3, IL-1β, pro-IL-1β, Caspase-1, and GSDMD-N. Post tanshinone IIA treatment, a noticeable reduction in the expression levels of these proteins was observed. It is acknowledged that upon NLRP3 activation, subsequent activation of Caspase-1 and GSDMD occurs, prompting the expression of IL-1β and IL-18, thereby intensifying the inflammatory response.

Research has indicated that tanshinone IIA has the potential to modulate the TLR4/NF-κB p65 signaling pathway, enhancing oxidative stress levels to address hypoxic/ischemic encephalopathy (Fang et al., 2018b). Simultaneously, it can suppress the TLR4/NF-κB pathway to mitigate the vulnerability of atherosclerotic plaques in ApoE^(−/−)^ mice (Wang et al., 2020c), thus showcasing its anti-inflammatory and immune-regulatory properties (Chen et al., 2019). Furthermore, tanshinone IIA has been shown to inhibit the inflammatory response of vascular smooth muscle cells induced by lipopolysaccharides (LPS) through the TLR4/TAK1/NF-κB signaling pathway (Meng et al., 2019). These collective findings suggest that tanshinone IIA can influence or partially activate the TLR4/NF-κB p65 signaling pathway. Building on these observations, we hypothesize that tanshinone IIA exerts its protective effects by mitigating cardiomyocyte pyroptosis, consequently reducing the inflammatory response associated with NLRP3 inflammasome activation. This study sheds light on the potential therapeutic role of tanshinone IIA in cardiac pathologies, offering insights into its underlying molecular mechanisms.

The TLR4/NF-κB pathway assumes a pivotal role in governing inflammation and pyroptosis. Activation of TLR4 triggers NF-κB activation, culminating in the transcription of pro-inflammatory cytokines and inflammasomes (like NLRP3), thereby instigating pyroptosis through Caspase-1. Numerous investigations have elucidated the significance of the TLR4/NF-κB pathway in pyroptosis. For example, the combined application of mangiferin and cinnamic acid has exhibited the capacity to impede TLR4/NLRP3-activated pyroptosis, consequently alleviating rheumatoid arthritis (Li et al., 2022). Similarly, in instances of liver I/R injury, both oxytocin and melatonin have been documented to mitigate inflammasome-induced pyroptosis via the TLR4/NF-κB/NLRP3 pathway (El-Sisi et al., 2021). Additionally, metformin interrupts the TLR4/NF-κB/PFKFB3 signal transduction, ameliorates abnormal glucose metabolism, and averts NLRP3 inflammasome-mediated pyroptosis (Zhang et al., 2021). These investigations collectively underscore the intimate association between the TLR4/NF-κB p65 signaling pathway and pyroptosis.

Increasing evidence suggests that excessive pyroptosis can lead to cardiac insufficiency, and targeting pyroptosis can improve heart function. Cathepsin B exacerbates diabetic cardiomyopathy by promoting NLRP3-mediated pyroptosis (Liu et al., 2022). By generating GSDMD global knockout mice, it has been found that GSDMD deficiency reduces Doxorubicin (Dox)-induced cardiomyopathy. Dox induces the activation of inflammatory caspases, which subsequently mediate the generation of GSDMD-N indirectly (Ye et al., 2022). The anti-inflammatory activity of apigenin inhibits pyroptosis through the NLRP3/Caspase1/Gasdermin D signaling axis, protecting the heart from LPS-induced cardiac dysfunction (Joshi et al., 2023). Cinnamic acid prevents myocardial I/R injury by inhibiting the NLRP3/Caspase-1/GSDMD signaling pathway (Luan et al., 2022). These studies collectively suggest that targeting pyroptosis can enhance heart function.

We acknowledge the limitations of our study. While we have proposed potential mechanisms underlying the inhibitory effect of tanshinone IIA on cardiomyocyte pyroptosis, we did not employ molecular manipulation techniques to thoroughly investigate these mechanisms. Our study lays the groundwork for future research. Subsequent studies could employ techniques like gene knockdown, overexpression, siRNA, or specific inhibitors to manipulate the expression or activity of key molecules in the suggested pathways. These approaches would aid in validating and elucidating the precise mechanisms by which tanshinone IIA exerts its inhibitory effects on cardiomyocyte pyroptosis. By addressing these limitations and conducting more comprehensive investigations, we can deepen our understanding of tanshinone IIA’s molecular mechanisms and bolster the scientific rationale for its potential therapeutic application in cardiac pathologies.

## 5 Conclusion

**FIGURE 6:**
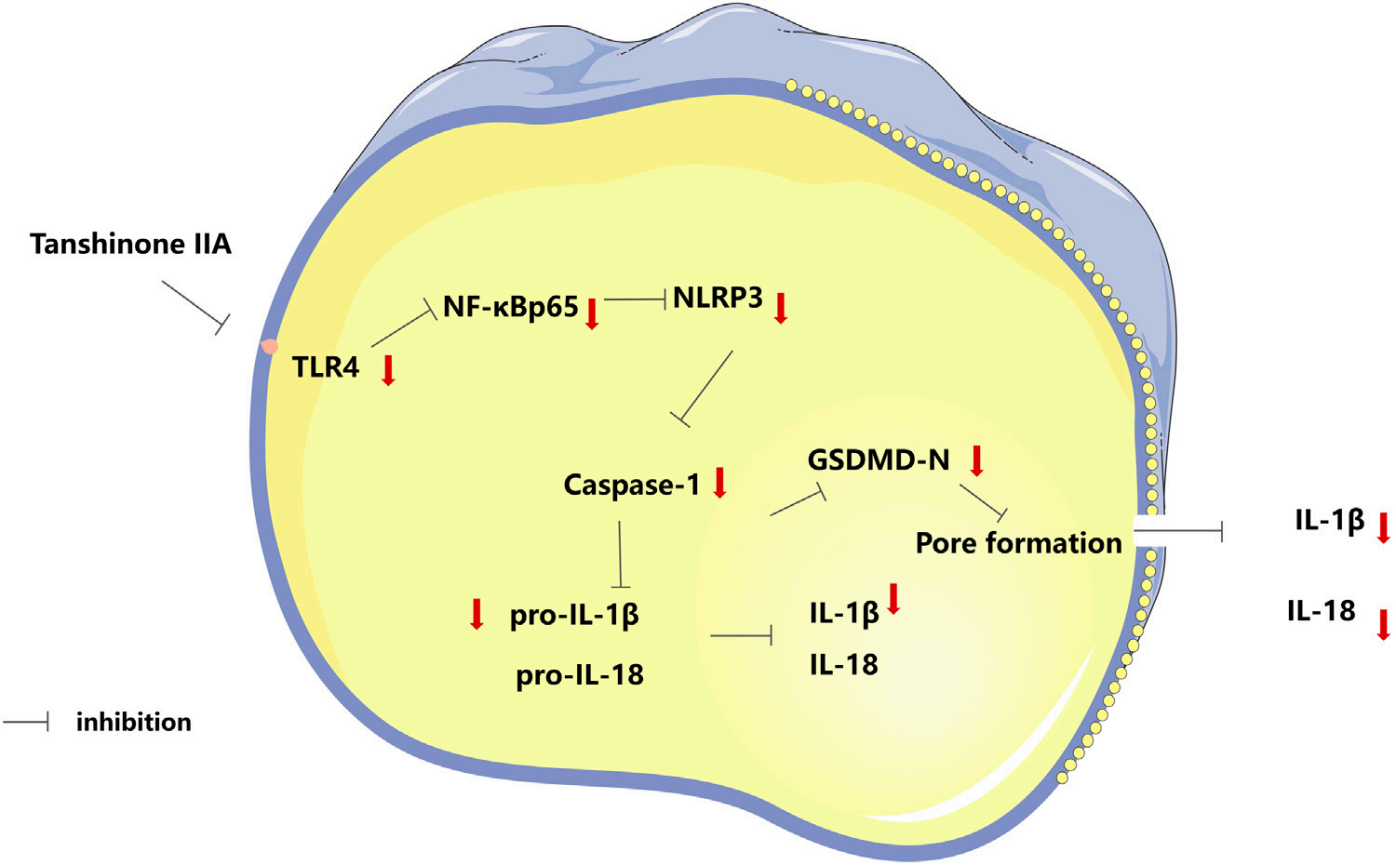
Tanshinone IIA protects cardiac function and inhibits cardiomyocyte pyroptosis through TLR4/NF-κBp65 pathway.

The findings suggested that tanshinone IIA ameliorates cardiac function and reduced myocardial injury in rats with HF after AMI. Furthermore, tanshinone IIA appeared to inhibit cardiomyocyte pyroptosis. The protective effect might be attributed to the inhibition of the TLR4/NF-κB p65 signaling pathway, which potentially presents a novel therapeutic target (Figure 6).

## Data Availability

The original contributions presented in the study are included in the article/Supplementary material, further inquiries can be directed to the corresponding authors.
